# Plant-Based Diets and Cognitive Outcomes: A Systematic Review and Meta-analysis

**DOI:** 10.1016/j.advnut.2025.100537

**Published:** 2025-10-16

**Authors:** Catherine Bigras, Riccardo Mazzoli, Danielle Laurin, Marcella Malavolti, Giulia Barbolini, Marco Vinceti, Jean-Philippe Drouin-Chartier, Tommaso Filippini

**Affiliations:** 1 Faculté de pharmacie, Université Laval, Québec, Canada; 2 Centre Nutrition, santé et société (NUTRISS), Université Laval, Québec, Canada; 3 Institut sur la nutrition et les aliments fonctionnels, Université Laval, Québec, Canada; 4 Centre de recherche du CHU de Québec- Université Laval, Axe Santé des populations et pratiques optimales en santé, Centre d’excellence sur le vieillissement de Québec, Québec, Canada; 5 CREAGEN - Environmental, Genetic and Nutritional Epidemiology Research Center, Section of Public Health, Department of Biomedical, Metabolic and Neural Sciences, University of Modena and Reggio Emilia, Modena, Italy; 6 Department of Epidemiology, Boston University School of Public Health, Boston, MA, United States; 7 School of Public Health, University of California Berkeley, Berkeley, CA, United States

**Keywords:** cognitive decline, cognitive impairment, dementia, vegetarian diet, plant-based diet

## Abstract

Although plant-rich dietary patterns like the Mediterranean and Mediterranean-DASH Intervention for Neurodegenerative Delay diets have been linked to cognitive benefits, the role of predominantly plant-based diets is less understood. This systematic review aimed to evaluate the association between plant-based diets and cognitive outcomes. A literature search was conducted in Medline and Embase using keywords related to plant-based diets (e.g., “vegetarian diet”) and cognitive outcomes (e.g., “dementia”). Studies of any design were eligible. Reviewers independently screened studies, extracted data, and assessed quality using the Newcastle-Ottawa Scale. Meta-analyses were conducted on prospective studies that examined the same dietary exposure and cognitive outcome, using fixed-effects regression models. Twenty-two studies were included, with considerable variability in methodologies and outcomes. Plant-based diets were defined either categorically (e.g., vegetarian compared with nonvegetarian), or using indices of adherence, such as the healthful plant-based diet index (hPDI), with higher scores reflecting higher adherence. Two meta-analyses, each based on 2 high-quality prospective cohort studies, examined associations between plant-based diet indices and cognitive outcomes. For cognitive impairment, pooled odds ratios (95% confidence interval) for highest compared with lowest quartiles were 0.61 (0.55, 0.68; *I*^*2*^ = 97.1%) for plant-based diet index (PDI) and 0.68 (0.62, 0.75; *I*^*2*^ = 84.3%) for hPDI. For dementia, pooled hazard ratios were 1.03 (0.91, 1.17; *I*^*2*^ = 0%) for PDI, 0.85 (0.75, 0.97; *I*^*2*^ = 0%) for hPDI, and 1.17 (1.03, 1.33; *I*^*2*^ = 60.3%) for unhealthful PDI. These findings suggest that dietary patterns emphasizing healthful plant-based foods and limiting less healthful plant foods and animal products are associated with lower odds of cognitive impairment and risk of dementia. However, findings across individual studies were inconsistent, highlighting the need for further high-quality research.

This review was registered at PROSPERO as CRD42022380055.

**Statement of significance**

This systematic review and meta-analysis suggests that dietary patterns emphasizing healthful plant-based foods and limiting less healthful plant foods and animal products are associated with lower odds of cognitive impairment and risk of dementia. However, findings across individual studies were inconsistent, highlighting the need for further high-quality research.

## Introduction

Dementia is a neurocognitive disorder that represents the advanced stage of a disease process that began 10–20 y before clinical manifestations [1]. Along the continuum, subtle cognitive changes can be detected using validated screening tools [2,3], neuropsychological test batteries [4] or medical examination. Cognitive impairment is an umbrella term referring to any unspecified impairment in cognitive function, whereas mild cognitive impairment (MCI) is a clinically defined condition in which deficits exceed those of normal aging but do not yet disrupt daily functioning [5]. With dementia now ranking as the seventh leading cause of death worldwide [6], its rising prevalence underscores the need for effective prevention strategies given the long latency period of the disease [7,8].

Diet is increasingly recognized as a modifiable lifestyle factor that may play a role in cognitive aging. The Mediterranean, Dietary Approaches to Stop Hypertension (DASH) and Mediterranean-DASH Intervention for Neurodegenerative Delay (MIND) diets are among the most studied dietary patterns in relation to cognitive health. All have been associated with slower rates of cognitive decline and a lower risk of dementia, supported by observational studies [[9], [10], [11], [12], [13]] and, in the case of the Mediterranean diet, interventional studies [14,15]. These diets emphasize the consumption of plant-based foods, such as fruits, vegetables, whole grains, legumes, nuts, and olive oil, while incorporating moderate amounts of animal products, particularly fish, and limiting red meat and saturated fat [16]. Despite being largely plant forward, these dietary patterns are not primarily defined as plant based. In contrast, predominantly plant-based diets, which include limited to no animal-derived foods, are less well studied in relation to cognitive outcomes [17].

Studying the impact of strict vegetarian or vegan diets on long-term health may be challenging in populations where the prevalence of these diets is low. The plant-based diet indices created by Satija et al. [18] offer a convenient way of studying the impact of increasing levels of adherence to a plant-based diet that can be applied to any population. The overall plant-based index (PDI) is derived by assigning positive scores to plant foods and reverse scores to animal foods. A healthful and unhealthful version of the PDI can be obtained by assigning positive scores to healthy plant foods and negative scores to unhealthy plant foods (hPDI) and conversely by assigning positive scores to unhealthy plant foods and negative scores to healthy plant foods (uPDI). The PDI indices have been studied in the context of various health outcomes [[19], [20], [21], [22]], but their association with the cognitive function is not yet understood.

Previous systematic reviews on plant-based diets and cognition have been limited in scope, with 1 identifying no eligible studies related to cognitive function [23], and another including only 2 studies on the topic [24]. Both reviews predate the emerging use of the PDI indices. We therefore conducted a systematic review and meta-analysis to address these gaps by providing a comprehensive up-to-date evaluation of the evidence on the relationship between plant-based diets and cognitive outcomes namely cognitive performance, cognitive decline, MCI, nonspecified cognitive impairment, or dementia.

## Methods

This systematic review and meta-analysis was conducted in accordance with the PRISMA guidelines [25]. The protocol was a priori registered on the international systematic review registry (PROSPERO registration no. CRD42022380055).

### Eligibility criteria

Due to the scarcity of existing systematic reviews on the topic of plant-based diets and cognitive function, we employed relatively broad selection criteria to capture as much relevant studies as possible. The current review included studies based on the following PECOS criteria:

Population (P): adults in any geographic location;

Exposure (E): adherence to a vegetarian, vegan or plant-based diet;

Comparator (C): compared with nonvegetarian diets or lower levels of adherence;

Outcome (O): cognitive outcomes (i.e., cognitive performance, cognitive decline, MCI, or a nonspecified cognitive impairment, dementia). These were defined as follows:

• Cognitive performance: cognitive functioning measured using standardized tests such as the Mini Mental State Exam (MMSE), Montreal Cognitive Assessment (MoCA), or other cognitive test batteries.
• Cognitive decline: a measurable change in cognitive performance over time.
• MCI: based on study-reported clinical diagnosis.
• Nonspecified cognitive impairment: study-defined cognitive dysfunction identified through cognitive testing but not formally classified as MCI or dementia.
• Dementia: clinical diagnosis according to standard criteria or medical records.

Study design (S): observational cohort, cross-sectional and case control studies, interventional studies, were all considered for inclusion.

The detailed PECOS criteria are presented in Supplemental Table 1.

Although dietary patterns such as the Mediterranean, DASH, and MIND diets are often considered plant based, we excluded studies focusing on these patterns, as our focus was specifically on diets that emphasize plant-based/vegetarian/vegan eating as a primary characteristic. Studies that assessed the exposure according to a posteriori dietary patterns derived from factor analysis were also excluded, as these patterns are arbitrarily labeled and often lack consistency across studies, making them difficult to compare meaningfully. We also did not include studies that involved plant-based diet interventions as part of multidomain interventions. We excluded studies that assessed subjective cognitive complaints or mortality due to dementia. Studies that did not report effect estimates were also excluded.

Studies derived from the same cohorts were included. Although these studies used overlapping participant samples, they reported varying results due to differences in participant selection criteria, dietary pattern classification methods, and adjustment factors. To maintain transparency and capture the full range of findings, each study was included in the qualitative synthesis with clear identification of the cohort source.

### Literature search and study selection

We performed a literature search in Embase and MEDLINE from inception up to 23 March, 2025, using keywords related to a plant-based diet and cognitive outcomes, e.g., [(vegetarian or vegan OR plant-based, etc.) AND (cognitive function OR cognitive decline OR dementia, etc.)]. The detailed search strategy used for each database can be found in Supplemental Table 2. No time limitation, language restrictions, or any other filters were set. An additional manual search was conducted by screening the references of the articles found. Titles and abstracts were independently screened for eligibility by 2 authors and validated by a third author. Disagreements were solved with the help of other reviewers.

### Data extraction

Data extraction was performed by 2 authors, with independent validation by a third author. The following data were extracted and organized into summary tables: name of author, year of publication, study design, location, sample size, age and biological sex of participants, follow-up if applicable, method to assess plant-based diet exposure, method to assess cognitive outcome, measures of association [beta-coefficients, hazard ratios (HRs), odds ratios (ORs), relative risks with their 95% confidence intervals (CIs) or SE], and adjustment factors. In case of missing information, the corresponding author of the article was contacted for clarification.

### Quality assessment

Study quality was assessed using the Newcastle-Ottawa Scale (NOS) [26] by 2 independent authors, with disagreements resolved by consensus following review by 3 other authors. The NOS includes evaluation of selection, comparability (including control for confounding), and outcome assessment. We adapted the scale for cross-sectional studies by attributing a score of zero to the 3 questions that were not applicable (outcome not present at baseline, follow-up duration, and follow-up rate), thereby accounting for the inherent biases associated with this study design. Studies could receive a maximum of 9 points: scores of ≥7–9 were considered as “good,” 4–6 as “fair,” and ≤ 3 as “poor.” The detailed description of the criteria used to score the included studies is presented in Supplemental Table 3.

### Data analysis

We conducted the meta-analyses in accordance with the Cochrane Handbook, using R software (version 4.4.2) and the “meta” package [27]. Only prospective studies were included. Meta-analyses were performed when ≥2 studies included evaluated the same dietary exposure to the same cognitive outcome. When multiple studies were based on the same cohort, we included only 1 in the main meta-analysis—selecting the study with the largest sample size, most comparable outcomes, and similar categorization of PDI scores (e.g., into quartiles or tertiles)—to respect the principle of independence between studies. When possible, sensitivity analyses were conducted by replacing the selected study with each of the other studies from the same cohort in separate meta-analyses. We used effect estimates obtained from fully adjusted models, as reported by the original studies.

When meta-analysis was feasible, forest plots were used to display the pooled risk estimates for cognitive outcomes, comparing the highest compared with lowest quantiles of adherence to a plant-based diet. When possible, we also conducted sensitivity meta-analyses using continuous estimates (e.g., risk per 10-point increment in hPDI). Given the limited number of studies in each analysis, we used fixed-effects models. Standard random-effects models perform poorly with few studies, as the between-study variance cannot be reliably estimated [28]. The difference between studies was evaluated with the *I*^*2*^ statistic. Given the small number of studies, *I*^*2*^ was used as an exploratory tool rather than a definitive indicator of heterogeneity. Assessment of publication bias was not possible due to the small number of studies, which limits the reliability of standard tests.

## Results

### Study selection

**FIGURE 1:**
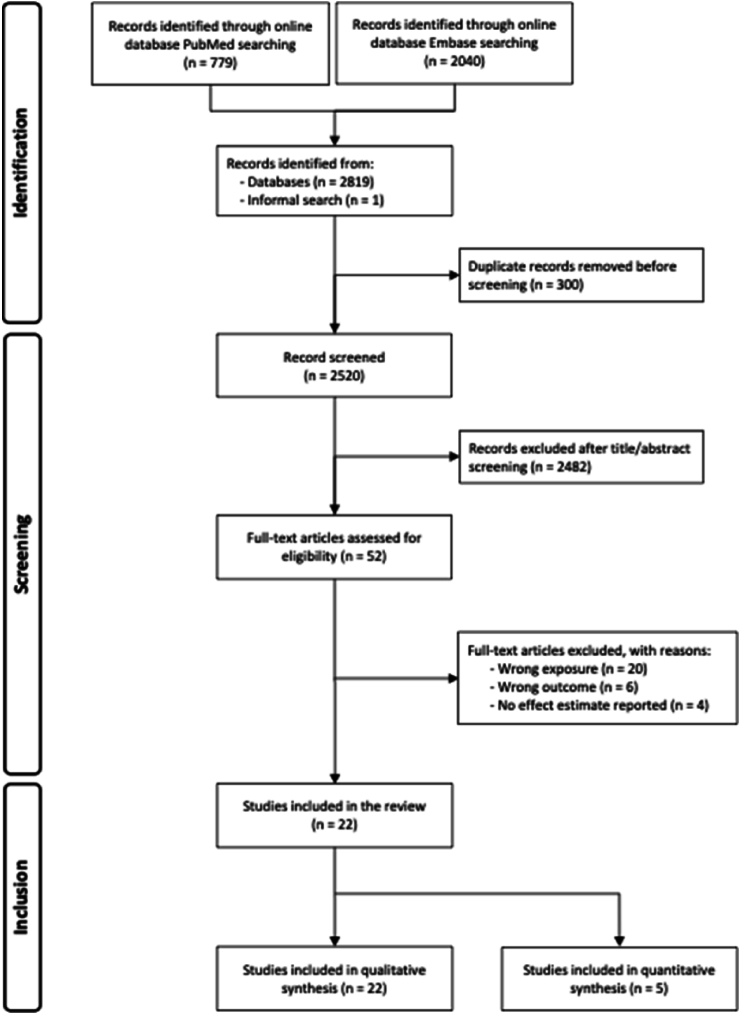
Flowchart of systematic literature search for studies that met the study inclusion and exclusion criteria.

A total of 2819 records were identified through database searches. One article was found outside the formal search strategy during the review process and included based on eligibility. After removal of duplicates, 2520 records were screened, of which 22 met the inclusion criteria and were selected for further analysis. Detailed information on the selection process is presented in Figure 1.

### Study characteristics

Descriptive characteristics of the studies included are presented in TABLE 1, TABLE 2. With 1 exception [29], all studies were published in the past 6 y. Of the 22 included studies, a total of 17 unique cohorts were represented in the review, as some studies were based on overlapping cohort data. All studies were observational: 15 were prospective cohort studies [29,30,32,34,[37], [38], [39], [40],[44], [45], [46], [47], [48], [49], [50]] (from 10 unique cohorts), 6 were cross-sectional [31,33,35,[41], [42], [43]] and 1 included both prospective and cross-sectional analyses [36]. No interventional studies were identified. Among the prospective cohort studies, age at baseline ranged from 53.3 to 83.8 y and follow-up ranged from 2 to 19.7 y. Most cohorts were from Asian countries (*n =* 9) followed by Europe (*n =* 4), and North America (*n =* 4). Dietary assessment varied among the studies, ranging from simple questionnaires (e.g., unspecified lifestyle questionnaires with few questions on diet) to 24 h diet recalls or food frequency questionnaires (FFQ). Classification of plant-based diet exposure also differed, with 7 studies comparing a vegetarian diet to a nonvegetarian diet in a dichotomous fashion [[29], [30], [31], [32], [33], [34], [35]], whereas 15 calculated increasing levels of plant-based diet adherence using indices such as the PDI, hPDI, and uPDI (from 10 unique cohorts) [[36], [37], [38], [39], [40], [41], [42], [43], [44], [45], [46], [47], [48], [49], [50]]. The outcomes analyzed included incidence of dementia (*n =* 7), prevalence of MCI or a nonspecified cognitive impairment (*n =* 12), and cognitive decline (*n =* 3). Cognitive impairment was assessed with the MMSE in 6 studies (3 unique cohorts), and MCI with the MoCA in 2 studies, whereas 4 studies relied on neuropsychological tests to assess cognitive impairment or decline. Table 3 presents the count of studies for each exposure-to-outcome combination.

**TABLE 1:** Characteristics of included studies that evaluated the association between strict vegetarian diets and cognitive outcomes (*n* = 7).

Cohort/study/population (Country)	Author (y)	Design	Sample size (*n*)	Age at baseline^1^ (y)	Follow-up	Dietary assessment method	Exposure	Cognitive outcome	Adjusted for	Results
Adventist Health Study-1 (United States)	Giem et al. (1993) [29]	Prospective- unmatched substudy	2984	>65	Study duration: 5 y	Lifestyle questionnaire including questions on past and current dietary habits	Meat > 4×/wk vs. vegetarian diet (no meat in 30 y) (ref.)	Probable dementia from hospital records	Age, sex, education	RR (95% CI): 0.86 (0.25, 2.94).
		Prospective- matched substudy	272	>65	Study duration: 5 y	Lifestyle questionnaire including questions on past and current dietary habits	Meat > 4×/wk vs. vegan and lacto-ovo-vegetarian diet (ref.)	Probable dementia from hospital records	Age, sex, education	RR = 2.18, *P =* 0.065.
Adventist Health Study-2 (United States + Canada)	Gatto et al. (2021) [30]	Prospective	132 (57.6% F)	75.1 ± 8.1^2^	Study duration: 10 y	>200-item quantitative FFQ	Vegetarian (vegan, lacto-ovo, pesco) vs. nonvegetarian diet (reference)	Mild memory impairment (MMI) based on comprehensive neuropsychological battery.	Age, education, ApoE genotype	OR (95% CI): 1.46 (0.51, 4.21).
Community-based sample among adults living in Pune (India)	Gazbare and Palekar (2024) [31]	Cross-sectional	605 (46.78% F)	49.21 ± 6.53	—	Lifestyle questionnaire (3 questions on diet-past 6 mo)	Vegetarian (only vegetables) vs. nonvegetarian diet (ref.)	MCI (based on ACE-III).	Age, gender, education, marital status, family type, BMI, sleep duration, stress, physical activity, lifestyle	OR (95% CI): 0.84 (0.52, 1.32).
HAICDDS Project (Taiwan)	Fan et al. (2023) [32]	Prospective	1285 (53% F)	72.4	2.58 y (mean)	Interview or review of medical chart to assess lifestyle factors including whether participants followed a vegetarian diet	Vegetarian vs. nonvegetarian diet (ref.)	Dementia (based on NIA-AA criteria and consensus meetings)	Age, gender, education, vascular risk factors (hypertension, diabetes, coronary artery disease, hypercholesterolemia, myocardial infarction), drugs (antihypertensives, anti-diabetics, antilipid agents), and lifestyle factors (smoking, alcohol).	HR (95% CI): 1.68 (1.03, 2.75)
Senior citizens in Kathmandu valley (Nepal)	Singh et al. (2021) [33]	Cross-sectional	304 (75.3% F)	≥60	—	Interview, no details on dietary assessment method	Nonvegetarian vs. lifetime vegetarian diet (ref.)	Dementia symptoms (based on 6-CIT).	Age, gender, education, past occupation, geriatric allowance, history of alcohol consumption, physical activity.	OR (95% CI): 2.31 (1.12, 4.76)
Tzu Chi Vegetarian Study (Taiwan)	Tsai et al. (2022) [34]	Prospective	4891 (73.5% F- veg group; 57.4% F- non veg-group)	58 ± 6.5 (veg group) 57.8 ± 6.3 (non-veg group)	9.2 y (mean)	Questionnaire, no details on dietary assessment method	Vegetarian (no meat, fish or poultry for ≥1 y) vs. nonvegetarian diet (ref.)	Dementia or mild cognitive impairment (based on ICD codes from death register data and medical claims records)	Age, sex, education, marital status, physical activity, smoking, alcohol, baseline medical comorbidities.	HR (95% CI): 0.671 (0.452, 0.996).
Women living in rural areas of Punjab (India)	Kaur and Kaur (2022) [35]	Cross-sectional	404 (100% F)	50.78 ± 8.1	—	Interview-based questionnaire, no details on dietary assessment method	Vegetarian vs. nonvegetarian diet (ref.)	Cognitive impairment (based on MMSE).	Study conducted among women only. Adjusted for age, education, age at menarche, marital status, physical activity, number of children, menopausal status.	OR (95% CI): 1.77 (1.02, 3.08).

Abbreviations: 6-CIT, 6-item Cognitive Impairment Test; AA, African American; ACE-III, Addenbrooke’s cognitive examination III; AFT, Animal Fluency Test; APOE ε 4, ε4 allele of the apolipoprotein E gene; CERAD, Consortium to Establish a Registry for Alzheimer’s Disease; CI, confidence interval; DSST, Digit Symbol Substitution Test; FFQ, food frequency questionnaire; GRS, genetic risk score; HR, hazard ratio; MCI, mild cognitive impairment; MMI, mild memory impairment; MMSE, Mini-Mental State Evaluation; MoCA, Montreal Cognitive Assessment; OR, odds ratio; PDI, hPDI and uPDI, overall, healthful and unhealthful plant-based diet indices; RAVLT, Rey Auditory Verbal Learning Test; RR, relative risk; SDMT, Symbol Digit Modalities Test; STICS-m, Spanish version of the modified Telephone Interview for Cognitive Status; TDI, Townsend deprivation index; HAICDDS, History-Based Artificial Intelligent Clinical Dementia Diagnostic System; NIA-AA, National Institute on Aging and Alzheimer's Association; ICD, International Classification of Disease.

1 Data presented as mean (mean ± SD) unless otherwise specified.

2 Age at cognitive testing.

**TABLE 2:** Characteristics of included studies that evaluated the association between plant-based diet adherence and cognitive outcomes (*n* = 15).

Cohort/study/population (Country)	Author (y)	Design	Sample size (*n*)	Age at baseline^1^ (y)	Follow-up	Dietary assessment method	Exposure	Cognitive outcome	Adjusted for	Results
B-vitamins for the Prevention of Osteoporotic Fractures (B-proof) trial (Netherlands)	van Soest etl al (2023) [36]	Cross-sectional	658 (41% F)	72.1 ± 5.4	—	190-item FFQ	PDI, hPDI, uPDI	Primary outcome: Global cognitive function (*Z*-scores), assessed using cognitive test battery (RAVLT, Digit Span task, Trail making test, Stroop test, SDMT, Letter fluency tests). Secondary outcome: domain-specific cognitive function (Z-scores) (episodic memory, attention and working memory, information processing speed, executive functioning).	Age, gender, education, BMI, physical activity, smoking, alcohol, margarine consumption.	βs (95% CI) per 10-point increment: Global cognitive function: PDI: 0.04 (–0.02, 0.10); hPDI: –0.02 (–0.08, 0.03); uPDI: –0.02 (–0.06, 0.05). Episodic memory PDI: 0.07 (–0.01, 0.16); hPDI: 0.00 (–0.08, 0.07); uPDI: 0.03 (–0.05, 0.11). Attention and working memory: PDI: –0.02 (–0.12, 0.08); hPDI: –0.06 (–0.15, 0.03); uPDI: 0.01 (–0.09, 0.10). Information processing speed PDI: 0.04 (–0.05, 0.13); hPDI: –0.03 (–0.11, 0.06); uPDI: 0.00 (–0.09, 0.09). Executive functioning PDI: 0.06 (–0.02, 0.15); hPDI: 0.01 (–0.07, 0.08); uPDI: –0.03 (–0.12, 0.05).
		Prospective	314	72.1 ± 5.4^2^	Study duration: 2 y	190-item FFQ	PDI, hPDI, uPDI	Primary outcome: change in global cognitive function (Z-scores), assessed using cognitive test battery (RAVLT, Digit Span task, Trail making test, Stroop test, SDMT, Letter fluency tests). Secondary outcome: change in Z-scores of domain-specific cognitive function (episodic memory, attention and working memory, information processing speed, executive functioning).	Age, gender, education, BMI, physical activity, smoking, alcohol, margarine consumption, baseline cognition score.	βs (95% CI) per 10-point increment: Change in global cognitive function: PDI: –0.03 (–0.08, 0.02); hPDI: 0.03 (–0.01, 0.08); uPDI: –0.05 (–0.09, 0.00). Change in episodic memory PDI: –0.06 (–0.17, 0.05); hPDI: 0.00 (–0.10, 0.10); uPDI: –0.04 (–0.14, –0.06). Change in attention and working memory: PDI: 0.04 (–0.08, 0.16); hPDI: 0.14 (0.03, 0.25); uPDI: –0.16 (–0.27, –0.05). Change in information processing speed: PDI: –0.07 (–0.16, 0.01); hPDI: 0.01 (–0.07, 0.09); uPDI: –0.07 (–0.16, 0.01). Change in executive functioning: PDI: –0.03 (–0.06, 0.11); hPDI: –0.01 (–0.09, 0.07); uPDI: 0.03 (–0.05, 0.12).
Chicago Health and Aging Study (United States)	Liu et al. (2022) [37]	Prospective	3337 (64% F)	73.7 ± 5.7	Study duration: 10 y	144-item semiquantitative FFQ	PDI, hPDI, uPDI	Primary outcome: cognitive decline (annual rate of decline in global cognition: composite score of immediate and delayed recall of East Boston Story (2 tests of episodic memory), SDMT (perceptual speed), and MMSE). Secondary outcomes: rate of decline in perceptual speed and episodic memory.	Results stratified by race: “White and African American.” Adjusted for age, sex, apoE e4 allele, education, calories, cognitive activities, smoking status, comorbidities (history of hypertension, diabetes, myocardial infarction, stroke), time, and their respective interactions with time.	βs ± SE, Q5 vs. Q1: African American participants*:* Global cognition PDI: 0.0072 ± 0.0073, *P =* 0.51 hPDI: 0.0183 ± 0.0086, *P =* 0.04 uPDI: –0.0137 ± 0.0086, *P =* 0.21 Perceptual speed PDI: 0.0113 ± 0.0075, *P =* 0.10 hPDI: 0.0179 ± 0.0088, *P =* 0.03 uPDI: –0.0083 ± 0.0088, *P =* 0.48 Episodic memory PDI: 0.0072 ± 0.0073, *P =* 0.51 hPDI: 0.0163 ± 0.0118, *P =* 0.04 uPDI: –0.0137 ± 0.0086, *P =* 0.21 White participants*:* Global cognition PDI: 0.0010 ± 0.0095, *P =* 0.24 hPDI: –0.0047 ± 0.0098, *P =* 0.65 uPDI: –0.0091 ± 0.0096, *P =* 0.44 Perceptual speed PDI: 0.0054 ± 0.0113, *P =* 0.80 hPDI: 0.0049 ± 0.0117, *P =* 0.67 uPDI: 0.0056 ± 0.0114, *P =* 0.66 Episodic memory PDI: 0.0010 ± 0.0095, *P =* 0.24 hPDI: –0.0047 ± 0.0098, *P =* 0.66 uPDI: –0.0091 ± 0.0096, *P =* 0.44
Chinese Longitudinal Healthy Longevity Survey (China)	Chen et al. (2025) [38]	Prospective	10,617 (50.7% F)	83.8 ± 10.8	4.9 y (mean)	15-item nonquantitative FFQ	uPDI	Cognitive impairment (based on MMSE)	Age, sex, education, married status, living pattern, exercise, smoking, drinking, household income, and BMI.	HRs (95% CI): per 1-unit increment: 1.02 (1.01, 1.03) T3 vs. T1 : 1.38 (1.18, 1.62).
	Liang et al. (2022) [39]	Prospective	4792 (49.4% F)	80.7 ± 9.58	24156 person-years (∼5.04 y /person)	16-item nonquantitative FFQ	PDI, hPDI, uPDI	Cognitive impairment (based on MMSE).	Sex, age, residence, education, occupation, smoking, alcohol consumption, physical activity, financial independence, health conditions.	HRs (95% CI): PDI < vs. > median: 1.32 (1.16, 1.50); hPDI < vs. > median: 1.46 (1.29, 1.66); uPDI > vs. < median: 1.21(1.06–1.38).
	Zhu et al. (2022) [40]	Prospective	6136 (46.33% F)	80 ± 9.83	Study duration: 10 y	16-item nonquantitative FFQ	PDI, hPDI, uPDI	Cognitive impairment (based on MMSE).	Age, sex, marital status, urban/rural residence, education, occupation before age 60, financial status, social and leisure activity, smoking and drinking status, physical activity, geographic regions, BMI, vitamin A/C/E intake, hypertension, diabetes, heart disease, cerebrovascular disease, and dyslipidemia.	ORs (95% CI), Q4 vs. Q1: PDI: 0.45 (0.39, 0.52); hDPI: 0.61 (0.54, 0.70); uPDI: 2.03 (1.79, 2.31).
Community-based Cohort Study on Nervous System Diseases (China)	He et al. (2025) [41]	Cross-sectional	1086 (52.7 % F)	≥55	—	81-item FFQ	PDI, hPDI, uPDI	MCI based on MoCA.	Results stratified by sex. For women: marital status, cereal and depression. For men: age, vegetable intake, vegetable oil intake and cereal.	OR (95% CI) for MCI among women: uPDI: 1.06 (1.02, 1.09). Men: NS, no result shown.
Middle-aged and elderly participants from Xiangyang city (China)	Peng et al. (2025) [42]	Cross-sectional	937 (66.9% F)	62.2 ± 9.2	—	Simplified FFQ	PDI, hPDI, uPDI	MCI based on MoCA test.	Age, sex, education, mean annual income, marital status, alcohol intake, smoking status, physical activity, BMI, heart disease, cerebrovascular disease, diabetes, hyperlipidemia, hypertension.	ORs (95% CI) for MCI, Q4 vs. Q1 : PDI: 0.82 (0.50, 1.35); hPDI: 0.80 (0.49, 1.30); uPDI: 2.21 (1.35, 3.60). ORs (95% CI) for MCI, per 10-point increase : PDI: 0.91 (0.66, 1.26); hPDI: 0.93 (0.66, 1.31); uPDI: 1.50 (1.15, 1.96).
NHANES (2011–2014) (United States)	Gong et al. (2025) [43]	Cross-sectional	2713 (51.4% F)^3^	59.8 ± 12.6	—	24 h recalls (≥1)	hPDI	Psychometric MCI (p-MCI) based on a neuropsychological test battery (CERAD World Learning Test, AFT, and DSST).	Age, sex, total energy intake, race, education, poverty-income ratio, smoking status, metabolic equivalent score, BMI, hypertension, diabetes and cardiovascular diseases.	ORs (95% CI) for p- MCI: T3 vs. T1 : 0.73 (0.54, 0.98). Per SD increment: 0.89 (0.78, 01.00).
Rotterdam Study (RS) (Netherlands)	de Crom et al. (2023) [44]	Prospective	9543 (58% F)	64.1 ± 8.6	14.5 y (mean)	170-item FFQ (subcohorts RS-1 and RS-11) and 389-item FFQ (subcohort RS-III)	PDI, hPDI, uPDI	Dementia (final diagnosis established by consensus panel according to DSM-III-R and NINCDS-ADRDA criteria).	Subcohort, age, sex, energy intake, education, alcohol, miscellaneous food intake, smoking, physical activity, APOE ε4 status, BMI, diabetes, total cholesterol, HDL cholesterol, use of lipid-lowering medication, systolic blood pressure, diastolic blood pressure, and use of blood-pressure lowering medication	HR (95% CI) for dementia, per 10-point increase: PDI: 0.99 (0.91, 1.08); hPDI: 0.93 (0.75, 1.01); uPDI: 1.02 (0.94, 1.10). HR (95% CI) for dementia, Q5 vs. Q1: PDI: 1.04 (0.87, 1.24); hPDI: 0.89 (0.74, 1.06); uPDI: 1.05 (0.87, 1.26).
Seguimiento Universidad de Navarra (SUN) cohort (Spain)	Munoz-Garcia et al. (2020) [45]	Prospective	806 (30.3% F)	61 ± 6	6 y (mean)	136-item semiquantitative FFQ	Provegetarian diet (PVD) score	Cognitive decline: mean 6-y change in STICS-m score (telephone adaptation of MMSE).	Age at baseline STICS, sex, follow-up time until baseline STICS-m, education, and APOE 4, smoking, package-years, total energy intake, physical activity, BMI, alcohol intake, depression, hypertension, high cholesterol, low HDL cholesterol, cardiovascular disease, type 2 diabetes.	β (95% CI) for 6-y change in STICS-m score per 6-point increase in PVD score = 0.19 (–0.03, 0.40).
Singapore Chinese Health Study (Singapore)	Wu et al. (2019) [46]	Prospective	16,948 (59.2% F)	53.5 ± 6.2	19.7 y (mean)	165-item semiquantitative FFQ	PDI, hPDI	Cognitive impairment (based on MMSE).	Age at cognitive status measurement, year of baseline interview, sex, dialect group, marital status, education level, smoking status, physical activity, sleep duration, BMI, total energy intake, alcohol consumption, baseline history of hypertension, diabetes, cardiovascular disease, and cancer.	ORs (95% CI) for cognitive impairment, Q4 vs. Q1: PDI: 0.82 (0.71, 0.94); hDPI: 0.78 (0.68, 0.90). ORs (95% CI) for cognitive impairment, per SD increment: PDI: 0.93 (0.88, 0.97); hDPI: 0.92 (0.88, 0.97).
	Zhou et al. (2021) [47]	Prospective	14,159 (59% F)	53.3 ± 6.1	∼20 y	165-item semiquantitative FFQ	PDI, hPDI	No cognitive impairment (based on MMSE).	Age at baseline, year of baseline interview, sex, dialect group, marital status, education, smoking status, physical activity, sleep duration, BMI, alcohol, and total energy intake.	ORs (95% CI) for no cognitive impairment, Q4 vs. Q1: PDI: 1.15 (0.99, 1.33); hDPI: 1.23 (1.06, 1.43). ORs (95%CI) for no cognitive impairment, per SD increment: PDI: 1.06 (1.00, 1.12); hPDI: 1.07 (1.02, 1.13).
United Kingdom Biobank (UK)	Shang et al. (2023) [48]	Prospective	115,093 (55.9% F)	59.0 ± 7.9	8.4–8.6 y (range)	24 h recalls (≥2)	hPDI	Dementia (based on ICD codes from hospital records and death register data).	Age, sex, and total energy intake, ethnicity, education, income, BMI, smoking, sleep, physical activity, and GRS for longevity.	HR (95% CI) for dementia, per quintile increment = 0.97 (0.92, 1.02).
	Wu et al. (2023) [49]	Prospective	180,532 (55% F)	Q3: 57 (median)	10 y (median)	24 h recalls (≥1)	PDI, hPDI and uPDI	Dementia (based on ICD codes from hospital records and death register data).	Age, sex, BMI, ethnicity, smoking status, alcohol, education, visiting friends, living alone, physical activity, total energy intake, medication history (use of antihypertensive, lipid-lowering and hypoglycemic medication), TDI, family history disease (dementia and depression), hypertension and diabetes.	HRs (95% CI) for dementia, per 10-point increase: PDI: 0.99 (0.90, 1.11); hPDI: 0.87 (0.79, 0.96); uPDI: 1.18 (1.07, 1.29). HRs (95% CI) for dementia, Q5 vs. Q1 : PDI: 1.03 (0.87, 1.23); hPDI: 0.82 (0.68, 0.98); uPDI: 1.29 (1.08, 1.53).
	Zhang et al. (2023) [50]	Prospective	114,684 (55.5% F)	56.8 ± 7.77	1,077,621 person-years (9.4 y /person)	24 h recalls (≥2)	PDI, hPDI, uPDI	Dementia (based on ICD codes from hospital records and death register data).	Age, sex, education, TDI, BMI, smoking status, alcohol consumption, regular physical activity, sleep duration, time on watching TV, family history of Alzheimer's disease, apolipoprotein E genotypes, cancer, cardiovascular disease, diabetes.	HR (95% CI) for dementia, T3 vs. T1 : PDI : 1.12 (0.90, 1.41). hPDI : 0.97 (0.77, 1.22). uPDI : 1.03 (0.82, 1.29).

Abbreviations: 6-CIT, 6-item Cognitive Impairment Test; AA, African American; ACE-III, Addenbrooke’s cognitive examination III; AFT, Animal Fluency Test; APOE ε 4, ε4 allele of the Apolipoprotein E gene; CERAD, Consortium to Establish a Registry for Alzheimer’s Disease; DSST, Digit Symbol Substitution Test; FFQ, food frequency questionnaire; GRS, genetic risk score; HR, hazard ratio; MCI, mild cognitive impairment; MMI, mild memory impairment; MMSE, Mini-Mental State Evaluation; MoCA, Montreal Cognitive Assessment; OR, odds ratio; PDI, hPDI and uPDI, overall, healthful and unhealthful plant-based diet indices; RAVLT, Rey Auditory Verbal Learning Test; RR, relative risk; SDMT, Symbol Digit Modalities Test; STICS-m, Spanish version of the modified Telephone Interview for Cognitive Status; TDI, Townsend deprivation index; ICD, International Classification of Disease; DSM-III-R, Diagnostic and Statistical Manual of Mental Disorders, Third Edition, Revised; NINCDS-ADRDA, National Institute of Neurological and Communicative Disorders and Stroke-Alzheimer's Disease and Related Disorders Association.

1 Data presented as mean (mean ± SD) unless otherwise specified.

2 Age at baseline of total sample.

3 Percent women of entire cohort.

**TABLE 3:** Number of included studies for each exposure-to-outcome combination.

Exposure	Outcomes		
	Cognitive performance or decline	Cognitive impairment/MCI	Dementia
Vegetarian diet vs. nonvegetarian diet	0	4 (MMSE: *n =* 1; ACE-III: *n =* 1; 6-CIT: *n =* 1; Neuropsychological tests: *n =* 1)	3 (Consensus panel, NIA-AA criteria: *n* = 1; hospital records: *n =* 1; ICD codes from death register and medical claims data: *n =* 1)
Plant-based diet indices (PDI, hPDI, uPDI, PVD)	3 (STICS-m: *n =* 1; Neuropsychological tests: *n =* 2)	8 (MMSE: *n =* 5; MoCA: *n =* 2; Neuropsychological tests: *n =* 1)	4 (Consensus panel, DSM-III-R and NINCDS-ADRDA criteria: *n =* 1; ICD codes from hospital records and death register data: *n =* 3)

Abbreviations: 6-CIT, 6-item Cognitive Impairment Test; ACE-III, Addenbrooke’s cognitive examination III; MCI, mild cognitive impairment; MMSE, Mini-Mental State Evaluation; MoCA, Montreal Cognitive Assessment; PDI, hPDI and uPDI, overall, healthful and unhealthful plant-based diet indices; PVD, provegetarian diet; STICS-m, Spanish version of the modified Telephone Interview for Cognitive Status; ICD, International Classification of Disease; DSM-III-R, Diagnostic and Statistical Manual of Mental Disorders, Third Edition, Revised; NINCDS-ADRDA, National Institute of Neurological and Communicative Disorders and Stroke-Alzheimer's Disease and Related Disorders Association.

Among the prospective cohort studies, 10 received good-quality ratings and 6 were rated fair. The 6 cross-sectional studies received fair scores apart from 1 that was rated as poor (Table 4) [[29], [30], [31], [32], [33], [34], [35], [36], [37], [38], [39], [40], [41], [42], [43], [44], [45], [46], [47], [48], [49], [50]].

**TABLE 4:** Assessment of study quality using the Newcastle-Ottawa Scale.

Study	Selection				Comparability	Outcome			Total score
	Representative-ness of the exposed cohort	Selection of the nonexposed cohort	Ascertainment of exposure	Outcome not present at start of study	Comparability of cohorts	Assessment of outcome	Length of follow-up	Adequacy of follow-up cohorts	
Giem et al. (1993) [29] 1	0	1	0	1	2	0	0	0	4
Giem et al. (1993) [29] 2	0	0	0	1	2	0	0	0	3
Gatto et al. (2021) [30]	0	1	1	0	1	1	1	0	5
Gazbare and Palekar (2024) [31]	1	1	0	0	2	1	0^3^	0^3^	5
Fan et al. (2023) [32]	0	1	0	1	2	1	0	1	6
Singh et al. (2021) [33]	0	1	0	0^3^	2	1	0^3^	0^3^	4
Tsai et al. (2022) [34]	0	1	0	1	2	1	0	1	6
Kaur and Kaur (2022) [35]	0	1	0	0^3^	2	1	0^3^	0^3^	4
van Soest et al. (2023) [36] 4	0	1	1	1	2	1	0	0	6
van Soest et al. (2023) [36] 5	0	1	1	0^3^	2	1	0^3^	0^3^	5
Liu et al. (2022) [37]	1	1	1	0	2	1	1	1	8
Chen et al. (2025) [38]	1	1	1	1	2	1	0	1	8
Liang et al. (2022) [39]	1	1	1	1	2	1	1	0	8
Zhu et al. (2022) [40]	1	1	1	1	2	1	1	1	9
He et al. (2025) [41]	1	1	0	0^3^	0	1	0^3^	0^3^	3
Peng et al. (2025) [42]	0	1	0	0^3^	2	1	0^3^	0^3^	4
Gong et al. (2025) [43]	1	1	1	0^3^	2	1	0^3^	0^3^	6
De Crom et al. (2023) [44]	1	1	1	1	2	1	1	1	9
Munoz-Garcia et al. (2020) [45]	0	1	1	1	2	1	0	0	6
Wu et al. (2019) [46]	1	1	1	0	2	1	1	1	8
Zhou et al. (2021) [47]	1	1	1	0	2	1	1	1	8
Shang et al. (2023) [48]	1	1	1	1	2	1	0	1	8
Wu et al. (2023) [49]	1	1	1	1	2	1	0	1	8
Zhang et al. (2023) [50]	1	1	1	1	2	1	0	1	8

1 Unmatched substudy.

2 Matched substudy.

3 Questions not applicable to cross-sectional studies

4 Longitudinal analysis.

5 Cross-sectional analysis.

### Summary of study findings

Overall, findings related to the association between plant-based diets and cognitive outcomes were inconsistent, with some studies reporting protective associations, whereas others found no evidence of association or even harmful associations (Table 1).

### Strict vegetarian diets and cognitive outcomes

The association between strict vegetarian diets and cognitive outcomes was examined in 4 prospective cohort studies [29,30,32,34] and 3 cross-sectional studies [31,33,35] (Table 1). The study populations varied widely, and the results were inconsistent across study designs and outcomes. Among the 4 prospective studies, 1 reported a beneficial association [34], 2 reported no association [29,30] and 1 suggested a detrimental association [32]. Similarly, among the 3 cross-sectional studies, 1 reported a beneficial association [33], 1 found no association [31], and 1 suggested a detrimental association [35]. All prospective and cross-sectional studies received low-to-fair quality ratings. It was not possible to conduct a meta-analysis because of the variability in the study populations, exposures and cognitive outcomes.

### Adherence to a plant-based dietary pattern and cognitive outcomes

A total of 12 prospective cohort studies [[36], [37], [38], [39], [40],[44], [45], [46], [47], [48], [49], [50]] and 3 cross-sectional studies [[41], [42], [43]] examined the association between adherence to a plant-based dietary pattern and cognitive outcomes (Table 2). Among the 12 prospective cohort studies, adherence to a plant-based diet was assessed using the plant-based diet indices (PDI, hPDI, uPDI) in 7 unique cohorts. One study used the provegetarian diet score (PVD) [45], which is similar to the PDI. Three cohort studies with follow-up duration ranging from 2 to 10 y measured cognitive change over time and reported no evidence of association [36,37,45], except for Liu et al. [37] who reported an inverse association between higher hPDI adherence and cognitive decline among African American participants but not white participants. Five studies, all conducted within 2 Asian cohorts with 10 and 19.7 y of follow-up, measured the association between the plant-based diet indices and cognitive impairment and suggested a beneficial association for highest adherence to the PDI and hPDI [[38], [39], [40],^46^,^47^]. Risk of dementia was assessed in 4 studies conducted within 2 European cohorts [44,[48], [49], [50]], with mixed results. Although no association was found between the plant-based diet indices and risk of dementia in the Rotterdam Study [44], results were mixed among the 3 studies based on UK Biobank cohort, with 2 studies reporting no significant association between the plant-based diet indices and risk of dementia [48,50], and 1 reporting a beneficial association for hPDI and a detrimental association for uPDI [49]. Ten out of the 12 prospective cohort studies received good-quality ratings and 2 were rated fair.

Among the 3 cross-sectional studies that measured the association between the plant-based diet indices and MCI, beneficial results were reported for the hPDI in 1 of the 2 studies that measured it [43], whereas detrimental results were found for the uPDI in the 2 studies that measured it [41,42]. The 3 cross-sectional studies received low-to-fair quality ratings.

### Meta-analyses results

Prospective studies with similar dietary exposures and outcomes were selected for meta-analysis. For duplicate cohorts, only 1 study was included in the main meta-analysis. Reasons for selecting and excluding duplicate cohorts for the meta-analyses are presented in Supplemental Table 4.

### Adherence to a plant-based diet and odds of cognitive impairment

**FIGURE 2:**
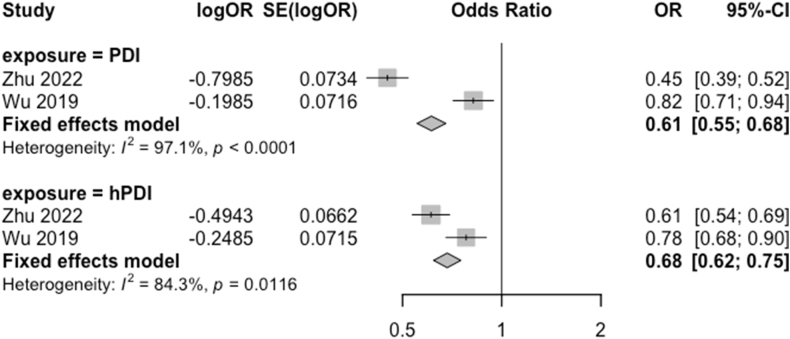
Association of each plant-based diet index (PDI) with odds of cognitive impairment, for high (quartile 4) vs. low (quartile 1) adherence to each plant-based diet pattern. Weights of each estimate are represented by the size of the square. The black lines represent the individual estimate effects (vertical), and the 95% confidence interval (CI). The *x*-axis is the odds ratio. The diamonds represent the pooled effect sizes and 95% CIs, estimated using fixed effect models. *I*^2^ refers to the proportion of heterogeneity between studies. The study by Zhu et al. [40] was conducted in the Chinese Longitudinal Healthy Longevity Survey, and the study by Wu et al. [46] was conducted in the Singapore Chinese Health Study. hPDI, healthful PDI.

The meta-analysis comparing the association between highest compared with lowest adherence to the PDI and hPDI and the odds of cognitive impairment included 2 Asian cohorts [40,46] and totaled 23,084 participants (Figure 2). Both studies received good-quality ratings. The pooled ORs (95% CI) for highest compared with lowest quartiles were 0.61 (0.55, 0.68; *I*^*2*^ = 97.1%) for PDI and 0.68 (0.62, 0.75; *I*^*2*^ = 84.3%) for hPDI. The uPDI was not calculated in 1 of the studies and could not therefore be included in the meta-analysis.

### Adherence to a plant-based diet and risk of dementia

**FIGURE 3:**
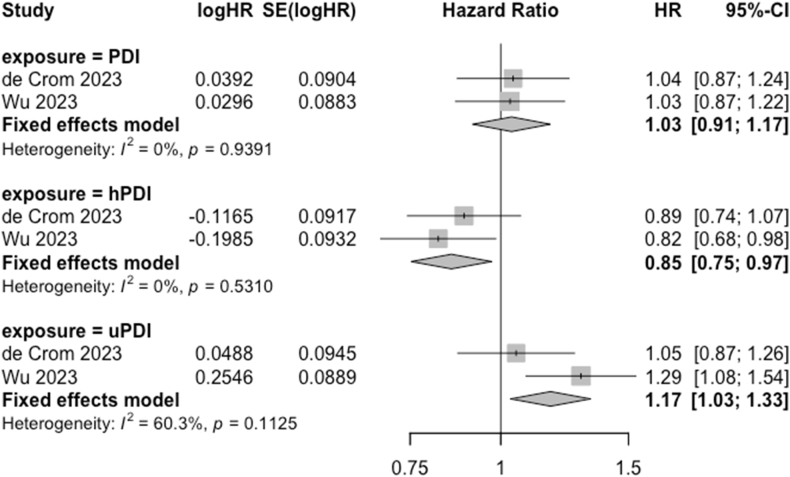
Association of each plant-based diet index (PDI) with dementia risk, for high (quintile 5) vs. low (quintile 1) adherence to each plant-based diet pattern. Weights of each estimate are represented by the size of the square. The black lines represent the individual estimate effects (vertical), and the 95% confidence interval (CI). The *x*-axis is the hazard ratio. The diamonds represent the pooled effect sizes and 95% CIs, estimated using fixed effect models. *I*^2^ refers to the proportion of heterogeneity between studies. The study by de Crom et al. [44] was conducted in the Rotterdam study, and the study by Wu et al. [46] was conducted in the United Kingdom Biobank. hPDI, healthful PDI; uPDI, unhealthful PDI.

The meta-analysis comparing the association between highest compared with lowest adherence to the PDI, hPDI, and uPDI and risk of dementia included 2 European cohorts [44,49] and totaled 190,075 participants (Figure 3). Both studies were rated as good-quality. The pooled HRs (95% CI) for the highest compared with lowest quintile were 1.03 (0.91, 1.17; *I*^*2*^ = 0%) for PDI, 0.85 (0.75, 0.97; *I*^*2*^ = 0%) for hPDI, and 1.17 (1.03, 1.33; *I*^*2*^ = 60.3%) for uPDI. A sensitivity analysis using the plant-based dietary indices continuously (per 10-point increment) rather than categorically yielded similar results (Supplemental Figure 1). Another sensitivity analysis was conducted by replacing the United Kingdom Biobank sample with an alternative study sample based on the same cohort [50]. In this study, participants were divided into tertiles of adherence rather than quintiles, and the HRs from the meta-analysis were no longer significant (Supplemental Figure 2).

## Discussion

This systematic review of 22 prospective and cross-sectional studies suggests a favorable association between adherence to plant-based diets and cognitive outcomes. Although variability in study populations, follow-up duration, dietary assessments and outcomes may explain some inconsistencies, in the 2 meta-analyses each including 2 good-quality prospective cohort studies on the association between adherence to a plant-based diet and cognitive outcomes, pooled results revealed that when compared with the lowest levels of adherence, highest hPDI adherence was associated with a modestly lower risk of both cognitive impairment and dementia. The results were mixed when adherence to a plant-based diet was considered without accounting for the quality of the plant foods (i.e., when the exposure was the PDI). Higher adherence to the PDI was associated with a lower risk of cognitive impairment, but no association was observed for dementia. However, the highest uPDI adherence was associated with a higher risk of dementia. Although these results are based on a limited number of studies, they provide initial evidence that a dietary pattern emphasizing healthy plant-based foods while limiting the consumption of unhealthy plant foods and animal is associated with a reduced risk of cognitive impairment and dementia.

The present study is an important addition to the body of meta-evidence on plant-based diets and cognitive outcomes. A 2021 systematic review and meta-analysis by Iguacel et al. [24] included only 2 studies on vegetarian diets and cognitive outcomes. The meta-analysis combined unadjusted data from 2 studies with different populations and outcomes and did not yield any significant effect. In contrast, our review includes a larger number of studies and provides initial estimates regarding the PDI indices based on large, good-quality cohort studies with extended follow-up periods.

The present review found inconsistent results across 7 prospective and cross-sectional studies examining the association between strict vegetarian and vegan diets and cognitive outcomes. Although some studies reported beneficial effects, others found no association or even suggested potential detrimental effects. However, all studies were given low or fair quality ratings and variability in study populations and methods likely contributed to the inconsistency in findings. One important limitation of a dichotomous classification of individuals as vegetarians or nonvegetarians is that it may not accurately reflect the complexity of dietary patterns. Vegetarian diets can vary widely in their inclusion of animal-derived foods, and do not necessarily reflect the healthfulness or overall quality of the dietary pattern, which could partly explain some of the inconsistent findings observed in this review.

The PDI scores provide a more comprehensive graded assessment of adherence to plant-based diets. Notably, findings on their association with cognitive outcomes were generally more consistent across studies employing similar measures, particularly among the 12 prospective studies. The meta-analyses included good-quality cohort studies and provided evidence for a beneficial association between increasing levels of adherence to a plant-based diet and cognitive outcomes. The hPDI, which emphasizes the consumption of healthy plant-based foods, was associated with a lower risk of both dementia and cognitive impairment. Conversely, the uPDI, which reflects a diet rich in unhealthy plant foods such as added sugars and refined grains, was associated with a higher risk of dementia. High uPDI scores could reflect high intakes of ultraprocessed foods, which has been linked to adverse cognitive outcomes in previous research [51,52]. Although the meta-analyses revealed promising results, they should be considered in light of the characteristics of the included studies. In the meta-analysis on the association between PDI/hPDI/uPDI scores and risk of dementia, both studies were conducted in European countries in populations of relatively similar ages (64.1 y; 57 y) and follow-up duration (14.5 y; 10 y), using detailed dietary assessments. *I*^*2*^ values were low for PDI and hPDI, and higher for uPDI. However, given the inclusion of only 2 studies, the *I*^*2*^ statistic may not reliably estimate between-study variability and should be considered as an exploratory indicator of heterogeneity [53]. Conversely, in the meta-analysis on the association between PDI/hPDI and cognitive impairment, whereas both studies were from Asian countries, the participants from Zhu et al. [40] were of advanced age at baseline (mean: 80 y) compared with the participants from Wu et al. [46] (mean: 53.5 y). The use of a brief 16-item FFQ in Zhu et al. [40] combined with differences in participant age may explain the considerable between-study variability reflected in the *I*^*2*^ statistic for PDI and hPDI that could limit the interpretation of the OR estimate for cognitive impairment. Nonetheless, the direction of the estimates was consistent across all meta-analyses, supporting the beneficial association of hPDI and detrimental association of uPDI with cognitive outcomes.

The results of the present systematic review fit in the larger body of literature supporting the cognitive health benefits of plant-rich dietary patterns such as the Mediterranean, DASH, and MIND diets [9,11,13]. Despite shared characteristics, the effectiveness of the hPDI relative to these other dietary patterns is unclear. A large-scale study using data from the Nurses’ Health Study and the Health Professionals Follow-Up Study comparing 8 different dietary patterns and their association with healthy aging found that although the hPDI was significantly associated with increased odds of reaching the age of 70 y old without impairments in cognitive, physical and mental health, it was the least strongly associated when compared with other dietary patterns [54]. Other dietary patterns that included small amounts of animal foods, such as the Planetary Health Diet Index, were more strongly associated with maintaining subjective intact cognitive health than the hPDI. Some studies included in the present review also compared the PDI and hPDI with different dietary patterns and their association with cognitive outcomes. Using data from the United Kingdom Biobank, Shang et al. [48] compared the association between the Alternate Mediterranean Diet score (aMED), Alternative Healthy Eating Index (AHEI)-2010, hPDI and Anti-Empirical Dietary Inflammatory Index (AEDII) dietary patterns and chronic diseases, and found that the aMED and AHEI-2010—but not the hPDI and AEDII—were significantly associated with a reduced risk of dementia. Wu et al. [46] used data from the Singapore Chinese Health Study and prospectively assessed the association between 5 dietary patterns and the odds of cognitive impairment. Although all dietary patterns were significantly associated with a reduced risk, the PDI, followed by the hPDI, were the least strongly associated. These findings suggest that the hPDI may be less effective in reducing risk of cognitive impairment and dementia compared with other healthy dietary patterns. Current literature on the association between animal food groups and brain health is mixed. Although red meat appears to be associated with negative outcomes [55], dairy products may have protective effects [56], and fish has been more consistently linked to cognitive benefits [57,58]. The hPDI might therefore not fully reflect an ideal dietary pattern for cognitive health and could explain some of the null associations observed in this review. For example, van Soest et al. [36] found that a higher hPDI was associated with slower rates of cognitive decline only in individuals who consumed >0.93 servings of fish per week. More research is needed to understand the effectiveness of the hPDI relative to other dietary patterns in the context of cognitive health.

Although the meta-analyses suggested beneficial associations, varying results were observed among other individual studies, sometimes across different subgroups. For example, in a prospective cohort study, Liu et al. [37] reported a significant inverse association between higher hPDI adherence and cognitive decline among African American participants but not white participants. Although De Crom et al. [44] reported no association between the PDI scores and dementia in the overall prospective cohort, significant inverse associations between higher hPDI scores and dementia were observed in subgroup analyses among men and apolipoprotein E e4 carriers. Although it was not possible to conduct subgroup analyses in this review, these findings suggest that plant-based diets may be more beneficial in some subgroups than others and should be explored further in future studies.

Study methodology may also account for some of the inconsistent findings. Dietary assessment methods ranged from brief, unvalidated questionnaires to comprehensive FFQs with hundreds of items. Simpler tools may lack the sensitivity to capture dietary patterns accurately, whereas detailed FFQs, though more informative, still rely on self-report dietary and are subject to recall bias, particularly in older populations. This can potentially attenuate or distort the association between diet and cognitive outcomes. Additionally, assessment of cognitive function varied widely, ranging from brief but validated screening tests to detailed neuropsychological test batteries, clinical evaluations or medical records. These measures capture different stages along the continuum and differ in their validity and reliability. In particular, screening tools such as the MoCA and MMSE are not diagnostic measures and may lead to misclassification [59]. These methodological issues limit outcome comparability and may potentially explain the inconsistent findings across studies. Additionally, as is common in longitudinal studies of aging populations, selection bias is possible, particularly due to self-selection or exclusion of more vulnerable individuals in older cohorts. This could explain some of the inconsistent findings in this review.

Strengths of this systematic review include its comprehensive scope and detailed search strategy. Although the meta-analyses were limited by the small number of eligible studies, the included studies were of good quality and featured large sample sizes, which supports the overall robustness of the findings. However, there are several limitations that should be acknowledged. The restricted number of studies combined with the substantial variability in dietary assessment methods and cognitive outcomes made quantitative synthesis challenging. It was not possible to assess publication bias, and assessment of heterogeneity was limited and unreliable due to the small number of studies included. Additionally, the meta-analyses relied on a fixed-effects model, which does not account for potential between-study variability and may constrain the generalizability of the findings. Moreover, all included studies were observational, which further limits the conclusions that can be drawn on the causal nature of the relationship between plant-based diets and cognitive health.

To the best of our knowledge, this is the first comprehensive systematic review and meta-analysis on the relationship between plant-based diets and cognitive outcomes, which included both strict vegetarian diets as well as assessment of graded adherence to plant-based diets. Specifically, it is the first to include the recently developed plant-based diet indices and offer initial estimates. Although current evidence is still limited, nearly all included studies were published in the past 6 y, suggesting that literature on this topic will likely continue to expand. This systematic review and meta-analysis provides a baseline for future investigations in this emerging research area.

In conclusion, although overall evidence was inconsistent, meta-analyses based on a subset of high-quality cohort studies suggest that a dietary pattern emphasizing healthy plant-based foods whereas limiting the consumption of unhealthy plant foods and animal foods is associated with a modestly lower risk of dementia and cognitive impairment. However, these results should be interpreted with caution, as they are based on a limited number of studies. Current evidence remains limited due to methodological variability across studies. More well-designed large-scale prospective studies with long follow-up durations are needed to clarify the relationship between plant-based diets and cognitive aging.

## Author contributions

GB, TF, MV designed the research; RM and TF performed the systematic literature search, RM, TF and CB screened publications for eligibility; CB and RM extracted the data and evaluated study quality, with validation from JPDC, DL and TF; CB performed the statistical analyses and drafted the manuscript. All authors read, revised and approved the final manuscript.

## Data availability

Data described in the manuscript, codebook, and analytic code will be made available on request pending application and approval.

## Funding

This study was supported by grants PRIN 2022 (no. 2022MHMRPR) and PRIN 2022 PNRR (no. P20229KSXB) from the Italian Ministry of University, and by grant FAR2023 from University of Modena and Reggio Emilia. J-PD-C is a research scholar of the Fonds de recherche du Québec – Santé.

## Conflict of interest

J-PD-C reports a relationship with Dairy Farmers of Canada that includes: consulting or advisory, funding grants, and speaking and lecture fees. J-PD-C reports a relationship with Weston Family Foundation that includes: funding grants. If there are other authors, they declare that they have no known competing financial interests or personal relationships that could have appeared to influence the work reported in this paper.
