# Supplementary material for: Plant-Based Diets and Cognitive Outcomes: A Systematic Review and Meta-analysis
Source: Adv Nutr. 2025 Oct 16;16(11):100537. doi: 10.1016/j.advnut.2025.100537 (PMC12637054; doi:10.1016/j.advnut.2025.100537)
Supplement: multimedia component 1 [file mmc1.docx]

**Plant-based diets and cognitive outcomes: a systematic review and meta-analysis.**

**Supplementary material**

Catherine Bigras, Riccardo Mazzoli, Danielle Laurin, Marcella Malavolti, Giulia Barbolini, Marco Vinceti, Jean-Philippe Drouin-Chartier, Tommaso Filippini

**Supplementary Table S1.** PECOS criteria for inclusion.

| **PECOS** | **Inclusion** |
| --- | --- |
| Population | Any kind of adult population. |
| Exposure | Adherence to a plant-based diet (vegan, vegetarian or increasing levels of adherence using plant-based dietary indices). |
| Comparison | Non plant-based dietary patterns. |
| Outcomes | Cognitive performance, cognitive decline, MCI, non-specificed cognitive impairment or dementia. |
| Study types | Observational (e.g., cohort, cross-sectional) and interventional. |

**Supplementary Table S2.** Literature database search.

| **Database** | **Literature search** |
| --- | --- |
| PubMed | (Memory Impairment OR Cognitive Dysfunctions OR Dysfunction, Cognitive OR Dysfunctions, Cognitive OR Cognitive Impairments OR Cognitive Impairment OR Impairment, Cognitive OR Impairments, Cognitive OR Mild Cognitive Impairment OR Cognitive Impairment, Mild OR Cognitive Impairments, Mild OR Impairment, Mild Cognitive OR Impairments, Mild Cognitive OR Mild Cognitive Impairments OR Mild Neurocognitive Disorder OR Disorder, Mild Neurocognitive OR Disorders, Mild Neurocognitive OR Mild Neurocognitive Disorders OR Neurocognitive Disorder, Mild OR Neurocognitive Disorders, Mild OR Cognitive Decline OR Cognitive Declines OR Decline, Cognitive OR Declines, Cognitive OR Mental Deterioration OR Deterioration, Mental OR Deteriorations, Mental OR Mental Deteriorations OR Dementias OR Amentia OR Amentias OR Senile Paranoid Dementia OR Dementias, Senile Paranoid OR Paranoid Dementia, Senile OR Paranoid Dementias, Senile OR Senile Paranoid Dementias OR Familial Dementia OR Dementia, Familial OR Dementias, Familial OR Familial Dementias) AND (Diets, Vegetarian OR Vegetarian Diets OR Vegetarian Diet OR Lacto-Vegetarian Diet OR Diet, Lacto-Vegetarian OR Diets, Lacto-Vegetarian OR Lacto Vegetarian Diet OR Lacto-Vegetarian Diets OR Plant-Based Diet OR Diets, Plant-Based OR Plant Based Diet OR Plant-Based Diets OR Diet, Plant-Based OR Diet, Plant Based OR Plant-Based Nutrition OR Nutrition, Plant-Based OR Plant Based Nutrition OR Lacto-Ovo Vegetarian Diet OR Diet, Lacto-Ovo Vegetarian OR Diets, Lacto-Ovo Vegetarian OR Lacto Ovo Vegetarian Diet OR Lacto-Ovo Vegetarian Diets OR Vegetarian Diet, Lacto-Ovo OR Vegetarian Diets, Lacto-Ovo OR Vegetarianism OR Vegan) |
| Embase | ('memory impairment'/exp OR 'memory impairment' OR (('memory'/exp OR memory) AND ('impairment'/exp OR impairment)) OR 'cognitive dysfunctions' OR (cognitive AND dysfunctions) OR 'dysfunction, cognitive' OR (dysfunction, AND cognitive) OR 'dysfunctions, cognitive' OR (dysfunctions, AND cognitive) OR 'cognitive impairments' OR (cognitive AND impairments) OR 'cognitive impairment'/exp OR 'cognitive impairment' OR (cognitive AND ('impairment'/exp OR impairment)) OR 'impairment, cognitive' OR (impairment, AND cognitive) OR 'impairments, cognitive' OR (impairments, AND cognitive) OR 'mild cognitive impairment'/exp OR 'mild cognitive impairment' OR (mild AND cognitive AND ('impairment'/exp OR impairment)) OR 'cognitive impairment, mild' OR (cognitive AND impairment, AND mild) OR 'cognitive impairments, mild' OR (cognitive AND impairments, AND mild) OR 'impairment, mild cognitive' OR (impairment, AND mild AND cognitive) OR 'impairments, mild cognitive' OR (impairments, AND mild AND cognitive) OR 'mild cognitive impairments' OR (mild AND cognitive AND impairments) OR 'mild neurocognitive disorder'/exp OR 'mild neurocognitive disorder' OR (mild AND neurocognitive AND ('disorder'/exp OR disorder)) OR 'disorder, mild neurocognitive' OR (('disorder,'/exp OR disorder,) AND mild AND neurocognitive) OR 'disorders, mild neurocognitive' OR (('disorders,'/exp OR disorders,) AND mild AND neurocognitive) OR 'mild neurocognitive disorders' OR (mild AND neurocognitive AND ('disorders'/exp OR disorders)) OR 'neurocognitive disorder, mild' OR (neurocognitive AND ('disorder,'/exp OR disorder,) AND mild) OR 'neurocognitive disorders, mild' OR (neurocognitive AND ('disorders,'/exp OR disorders,) AND mild) OR 'cognitive decline'/exp OR 'cognitive decline' OR (cognitive AND ('decline'/exp OR decline)) OR 'cognitive declines' OR (cognitive AND declines) OR 'decline, cognitive' OR (('decline,'/exp OR decline,) AND cognitive) OR 'declines, cognitive' OR (declines, AND cognitive) OR 'mental deterioration'/exp OR 'mental deterioration' OR (mental AND ('deterioration'/exp OR deterioration)) OR 'deterioration, mental' OR (('deterioration,'/exp OR deterioration,) AND mental) OR 'deteriorations, mental' OR (deteriorations, AND mental) OR 'mental deteriorations' OR (mental AND deteriorations) OR dementias OR 'amentia'/exp OR amentia OR amentias OR 'senile paranoid dementia' OR (senile AND ('paranoid'/exp OR paranoid) AND ('dementia'/exp OR dementia)) OR 'dementias, senile paranoid' OR (dementias, AND senile AND ('paranoid'/exp OR paranoid)) OR 'paranoid dementia, senile' OR (('paranoid'/exp OR paranoid) AND ('dementia,'/exp OR dementia,) AND senile) OR 'paranoid dementias, senile' OR (('paranoid'/exp OR paranoid) AND dementias, AND senile) OR 'senile paranoid dementias' OR (senile AND ('paranoid'/exp OR paranoid) AND dementias) OR 'familial dementia' OR (familial AND ('dementia'/exp OR dementia)) OR 'dementia, familial' OR (('dementia,'/exp OR dementia,) AND familial) OR 'dementias, familial' OR (dementias, AND familial) OR 'familial dementias' OR (familial AND dementias)) AND ('diets, vegetarian' OR (diets, AND ('vegetarian'/exp OR vegetarian)) OR 'vegetarian diets' OR (('vegetarian'/exp OR vegetarian) AND ('diets'/exp OR diets)) OR 'vegetarian diet'/exp OR 'vegetarian diet' OR (('vegetarian'/exp OR vegetarian) AND ('diet'/exp OR diet)) OR 'lacto-vegetarian diet'/exp OR 'lacto-vegetarian diet' OR (('lacto vegetarian'/exp OR 'lacto vegetarian') AND ('diet'/exp OR diet)) OR 'diet, lacto-vegetarian' OR (('diet,'/exp OR diet,) AND ('lacto vegetarian'/exp OR 'lacto vegetarian')) OR 'diets, lacto-vegetarian' OR (diets, AND ('lacto vegetarian'/exp OR 'lacto vegetarian')) OR 'lacto vegetarian diet'/exp OR 'lacto vegetarian diet' OR (lacto AND ('vegetarian'/exp OR vegetarian) AND ('diet'/exp OR diet)) OR 'lacto-vegetarian diets' OR (('lacto vegetarian'/exp OR 'lacto vegetarian') AND ('diets'/exp OR diets)) OR 'plant-based diet'/exp OR 'plant-based diet' OR ('plant based' AND ('diet'/exp OR diet)) OR 'diets, plant-based' OR (diets, AND 'plant based') OR 'plant based diet'/exp OR 'plant based diet' OR (('plant'/exp OR plant) AND based AND ('diet'/exp OR diet)) OR 'plant-based diets' OR ('plant based' AND ('diets'/exp OR diets)) OR 'diet, plant-based' OR (('diet,'/exp OR diet,) AND 'plant based') OR 'diet, plant based' OR (('diet,'/exp OR diet,) AND ('plant'/exp OR plant) AND based) OR 'plant-based nutrition' OR ('plant based' AND ('nutrition'/exp OR nutrition)) OR 'nutrition, plant-based' OR (('nutrition,'/exp OR nutrition,) AND 'plant based') OR 'plant based nutrition' OR (('plant'/exp OR plant) AND based AND ('nutrition'/exp OR nutrition)) OR 'lacto-ovo vegetarian diet'/exp OR 'lacto-ovo vegetarian diet' OR ('lacto ovo' AND ('vegetarian'/exp OR vegetarian) AND ('diet'/exp OR diet)) OR 'diet, lacto-ovo vegetarian' OR (('diet,'/exp OR diet,) AND 'lacto ovo' AND ('vegetarian'/exp OR vegetarian)) OR 'diets, lacto-ovo vegetarian' OR (diets, AND 'lacto ovo' AND ('vegetarian'/exp OR vegetarian)) OR 'lacto ovo vegetarian diet'/exp OR 'lacto ovo vegetarian diet' OR (lacto AND ovo AND ('vegetarian'/exp OR vegetarian) AND ('diet'/exp OR diet)) OR 'lacto-ovo vegetarian diets' OR ('lacto ovo' AND ('vegetarian'/exp OR vegetarian) AND ('diets'/exp OR diets)) OR 'vegetarian diet, lacto-ovo' OR (('vegetarian'/exp OR vegetarian) AND ('diet,'/exp OR diet,) AND 'lacto ovo') OR 'vegetarian diets, lacto-ovo' OR (('vegetarian'/exp OR vegetarian) AND diets, AND 'lacto ovo') OR 'vegetarianism'/exp OR vegetarianism OR 'vegan'/exp OR vegan) AND ('article'/it OR 'article in press'/it OR 'chapter'/it OR 'conference abstract'/it OR 'conference paper'/it OR 'conference review'/it OR 'editorial'/it OR 'erratum'/it OR 'letter'/it OR 'note'/it OR 'preprint'/it OR 'short survey'/it OR 'tombstone'/it) |

**Supplementary Table S3.** Description of the criteria used to score the quality of each included study.

| **Selection**  1) Representativeness of the exposed cohort  a) truly representative of the average adult general population in the community ✓  b) somewhat representative of the average adult general population in the community ✓  c) selected group of users (eg nurses, volunteers)  d) no description of the derivation of the cohort  2) Selection of the non exposed cohort  a) drawn from the same community as the exposed cohort ✓  b) drawn from a different source  c) no description of the derivation of the non exposed cohort  3) Ascertainment of exposure  a) a priori defined (e.g., hPDI) plant-rich dietary patterns, calculated from dietary data obtained from a validated method (24-h diet recall, FFQ, food diary) ✓  b) self-report of adherence to plant-rich diet, not cross-validated from diet data obtained using a validated method as defined above (e.g., «are you vegetarian?»), no description  4) Demonstration that outcome of interest was not present at start of study  a) yes, not cognitively impaired participants at baseline (based on screening or clinical evaluation) ✓  b) no (no statement on exclusion of cognitively impaired individuals at baseline) | **Comparability**  1) Comparability of cohorts on the basis of the design or analysis (max 2 stars)  a) study controls for education and age ✓  b) study controls for additional factors, namely sex or gender ✓ |
| --- | --- |
|  | **Outcome**  1) Assessment of outcome  a) for incidence of dementia: based on clinical diagnosis according to published criteria or medical records (ICD codes) using validated databases; ✓  b) for incidence of MCI: based on clinical diagnosis according to published criteria  or validated neurocognitive test batteries; ✓  c) for incidence of cognitive impairment/decline: use of validated cognitive tests; ✓  d) assessment via unvalidated tests or not based on international guidelines  e) self-report, or no info on outcome assessment specified  2) Was follow-up long enough for outcomes to occur  a) yes ✓  - cognitive impairment/dementia: age 75 and older=5 years; age 65-74=10 years; age 50-65 = >10 years  - cognitive decline: age 75 and older = 2 years; age 65-74 = 4 years.  b) no  3) Adequacy of follow up of cohorts  a) complete follow up - all subjects accounted for ✓  b) subjects lost to follow up unlikely to introduce bias (small number lost: less than 20% follow up, or description provided of those lost) ✓  c) follow up rate <80% and no description of those lost  d) no statement |

**Supplementary Table S4.** Reasons for selecting and excluding duplicate cohorts for meta-analyses

| Cohort | Study | Reasons for selection/exclusion |
| --- | --- | --- |
| Chinese Longitudinal Healthy Longevity Survey | Chen 2025 | Excluded: only measured uPDI, did not measure PDI and hPDI. |
|  | Liang 2022 | Excluded: smaller sample size, and shorter follow-up (5y vs. 10y for the other studies of the same cohort). |
|  | **Zhu 2022** | **Selected for main analysis:** measured PDI/hPDI/uPDI in quartiles and present OR which makes it comparable to other study. |
| Singapore Chinese Health Study | **Wu 2019** | **Selected for main analysis:** larger sample size and outcome is directly comparable to other study. |
|  | Zhou 2021 | Excluded: smaller sample size and outcome is reversed (no cognitive impairment). |
| UK Biobank | Shang 2023 | Excluded: shorter follow-up period and reports HR for each quintile increment which limits comparability with other studies. |
|  | **Wu 2023** | **Selected for main analysis:** largest sample size, measured PDI/hPDI/uPDI adherence in quintiles. |
|  | **Zhang 2023** | **Selected for sensitivity analysis:** hPDI adherence in tertiles, included participants with 2 or more 24h recalls. |

**
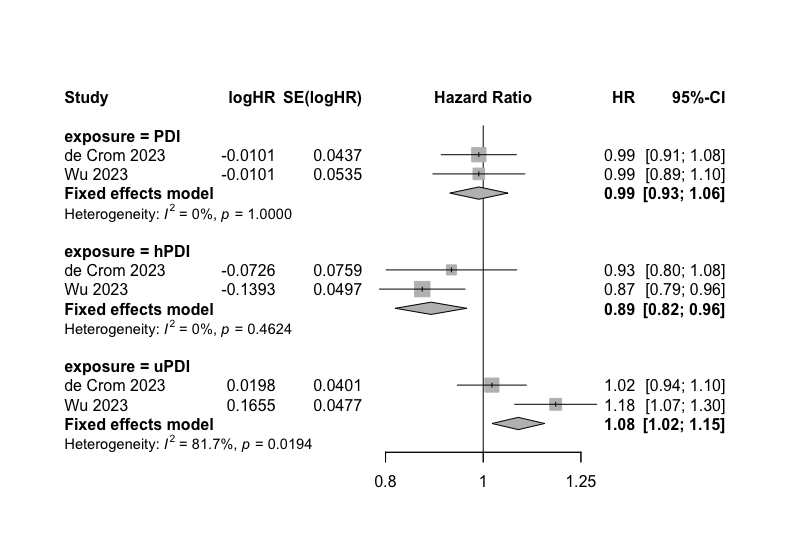
Supplementary Figure S1: Association of each plant-based diet index (PDI) with dementia risk, for each 10-point increment in each score**. Weights of each estimate are represented by the size of the square. The black lines represent the individual estimate effects (vertical), and the 95% CI. The *x*-axis is the hazard ratio. The diamonds represent the pooled effect sizes and 95% CIs, estimated using fixed effect models. *I*^2^ refers to the proportion of heterogeneity between studies. The study by de Crom (2023) was conducted in the Rotterdam study, and the study by Wu (2023) was conducted in the UK Biobank.

**
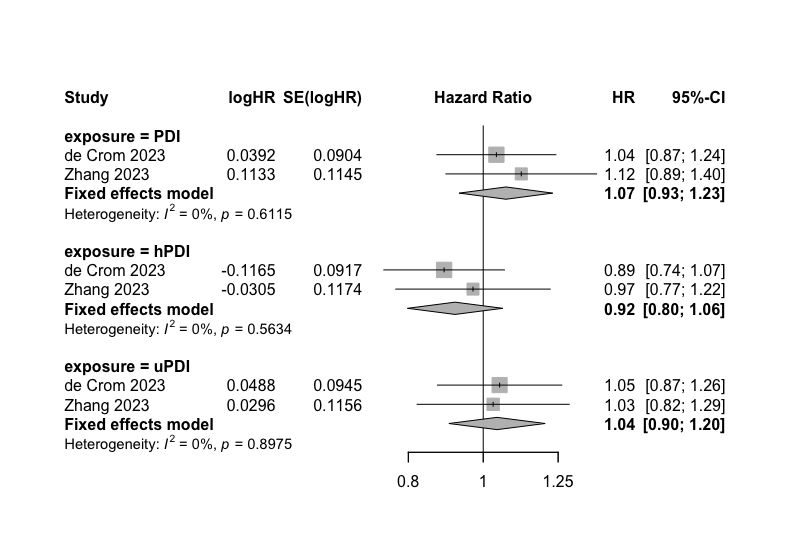
Supplementary Figure S2: Association of each plant-based diet index (PDI) with dementia risk, for high compared with low adherence to each plant-based diet pattern**. Weights of each estimate are represented by the size of the square. The black lines represent the individual estimate effects (vertical), and the 95% CI. The *x*-axis is the hazard ratio. The diamonds represent the pooled effect sizes and 95% CIs, estimated using fixed effect models. *I*^2^ refers to the proportion of heterogeneity between studies. The study by de Crom (2023) was conducted in the Rotterdam study. Risk estimates included in the meta-analysis for this study referred to quintile 5 vs quintile 1. The study by Zhang (2023) was conducted in the UK Biobank. Risk estimates included in the meta-analysis for this study referred to tertile 3 vs tertile 1.
